# Population Attributable Fractions Associated with Alzheimer’s Disease Risk Factors

**DOI:** 10.1093/geroni/igaf122.2529

**Published:** 2025-12-31

**Authors:** Igor Akushevich, Arseniy Yashkin, Svetlana Ukraintseva, Anatoliy Yashin, Julia Kravchenko

**Affiliations:** Duke University, Durham, North Carolina, United States; Duke University, Durham, North Carolina, United States; Duke University, Durham, North Carolina, United States; Duke University, Durham, North Carolina, United States; Duke University School of Medicine, Durham, North Carolina, United States

## Abstract

Although numerous risk factors for Alzheimer’s disease (AD) and related dementias (ADRD) have been identified, their combined influence on risk remains uncertain. In this study, we leverage a high-power 5%-sample of the Medicare population to assess the joint impact of AD/ADRD risk-related diseases and low income—as indicated by dual Medicare eligibility—on the risk of clinical diagnosis of AD/ADRD. We leveraged the univariable and multivariable Cox models for estimating AD/ADRD risks, identified most powerful predictors, and constructed predictive multivariable models for AD/ADRD risks, and evaluated their population attributable fractions (PAFs) for the general populations of older adults and race- and sex-specific subpopulations. The identified model included nine diseases—heart failure, hypertension, arrhythmia, stroke, hypotension, renal disease, depression, traumatic brain injury, and diabetes mellitus—as primary determinants of variation in AD/ADRD risk. The fraction of the total PAF explained by the predictors increased with age. The decomposition of the total PAF in terms of disease-specific PAFs showed that hypertension, stroke, and depression provided the strongest contribution for all subpopulations: Hypertension reached 45-50% of total PAF for Black, Asian, and Hispanic subpopulations. Stroke was the primary contributor for males (>30%); depression was highest among females and White individuals (approximately 30%). Hypertension and depression were the leading contributors for Native Americans (both approximately 30%). Heart Failure was the fourth strongest contributor for all subpopulations, with the contribution exceeding 5% for Native American individuals. Additional noteworthy contributors with PAFs exceeding 3% included hypotension (males), diabetes (Asian, Hispanic), arrhythmia (males), and TBI (Black, Asian).

